# Diagnosis of calcium pyrophosphate crystal deposition disease by ultrasonography: how many and which sites should be scanned?

**DOI:** 10.1093/rheumatology/kead565

**Published:** 2023-10-26

**Authors:** Edoardo Cipolletta, Erica Moscioni, Silvia Sirotti, Jacopo Di Battista, Abhishek Abhishek, Davide Rozza, Anna Zanetti, Greta Carrara, Carlo Alberto Scirè, Walter Grassi, Georgios Filippou, Emilio Filippucci

**Affiliations:** Rheumatology Unit, Department of Clinical and Molecular Sciences, Polytechnic University of Marche, Ancona, Italy; Academic Rheumatology, Injury, Recovery and Inflammation Sciences Department, School of Medicine, University of Nottingham, Nottingham, UK; Rheumatology Unit, Department of Clinical and Molecular Sciences, Polytechnic University of Marche, Ancona, Italy; Department of Rheumatology, IRCCS Galeazzi—Sant’Ambrogio Hospital, Milan, Italy; Rheumatology Unit, Department of Clinical and Molecular Sciences, Polytechnic University of Marche, Ancona, Italy; Academic Rheumatology, Injury, Recovery and Inflammation Sciences Department, School of Medicine, University of Nottingham, Nottingham, UK; Epidemiology Unit, Italian Society of Rheumatology, Milan, Italy; Epidemiology Unit, Italian Society of Rheumatology, Milan, Italy; Epidemiology Unit, Italian Society of Rheumatology, Milan, Italy; Epidemiology Unit, Italian Society of Rheumatology, Milan, Italy; Rheumatology Unit, Department of Clinical and Molecular Sciences, Polytechnic University of Marche, Ancona, Italy; Department of Rheumatology, IRCCS Galeazzi—Sant’Ambrogio Hospital, Milan, Italy; Rheumatology Unit, Department of Clinical and Molecular Sciences, Polytechnic University of Marche, Ancona, Italy

**Keywords:** chondrocalcinosis, CPPD, ultrasound, crystal arthritis, diagnostic accuracy

## Abstract

**Objective:**

To develop the optimal US scanning protocol for the diagnosis of calcium pyrophosphate crystal deposition (CPPD) disease.

**Methods:**

In this cross-sectional study, consecutive patients with a crystal-proven diagnosis of CPPD disease, and age-, sex-matched disease controls with a negative synovial fluid analysis were prospectively enrolled in two Italian Institutions. Four rheumatologists, blinded to patients’ clinical details, performed US examinations using a standardized scanning protocol including 20 joints (shoulders, elbows, wrists, metacarpophalangeal joints from second to fifth fingers, hips, knees, ankles). CPPD was identified as presence/absence, according to the OMERACT definitions. Reduced US scanning protocols were developed by selecting the most informative joints to be imaged by US using the LASSO technique. Patients were randomly divided into training and validation sets. Their diagnostic accuracy was tested comparing the area under the receiver operating characteristic curves.

**Results:**

The number of participants enrolled was 204: 102 with CPPD disease and 102 disease controls [age, mean (s.d.): 71.3 (12.0) *vs* 71.1 (13.5) years; female: 62.8% *vs* 57.8%]. The median number of joints with US evidence of CPPD was 5 [interquartile range (IQR): 4–7] and 0 (IQR: 0–1) in patients with CPPD disease and controls, respectively (*P* < 0.01). The detection of CPPD in ≥2 joints using a reduced scanning protocol (bilateral assessment of knees, wrists and hips) showed a sensitivity of 96.7% (95% CI: 82.8, 99.9) and a specificity of 100 (95% CI: 88.8, 100.0) for the diagnosis of CPPD disease and had good feasibility [mean (s.d.): 12.5 (5.3) min].

**Conclusion:**

Bilateral US assessment of knees, wrists and hips had excellent accuracy and good feasibility for the diagnosis of CPPD disease.

Rheumatology key messages

- Nearly 75% of patients with CPPD disease and none of disease controls had ≥4 joints with CPPD in an extended US scanning protocol of 20 joints.
- Knees, wrists and hips should always be included in US scanning protocols for CPPD; the US assessment of other joints should be considered in specific circumstances.
- A reduced six-joint US scanning protocol showed excellent accuracy (both sensitivity and specificity >90%) and good feasibility for the diagnosis of CPPD disease.

## Introduction

Calcium pyrophosphate deposition (CPPD) disease is a crystal arthropathy caused by the deposition of calcium pyrophosphate (CPP) crystals in and around the joint [1]. It is a common symptomatic arthropathy with a reported population prevalence ranging from 0.4% (i.e. those with a symptomatic CPPD disease) to 13.2% (i.e. those with radiographic evidence of chondrocalcinosis) [2, 3]. Moreover, CPPD disease is both a frequent primary cause of hospitalization and a common complication in people admitted to hospitals with another illness [4, 5].

The gold standard for the diagnosis of CPPD disease is the identification of CPP crystals in the synovial fluid (SF) by compensated polarized light microscopy, or occasionally, in biopsied tissues [6]. However, synovial fluid aspiration and/or analysis may not be always feasible (e.g. if small joints are affected or during inter-critical phases). Moreover, the absence of CPP crystal at synovial fluid analysis does not preclude a diagnosis of CPPD disease, as some studies have highlighted its suboptimal sensitivity in the detection of CPP crystals when compared with histology [7, 8]. Therefore, imaging techniques have a key role in clinical practice and in research, especially when laboratory evidence of CPP crystals is lacking [9]. In the last decade, ultrasonography (US) has emerged as one of the most valid, accurate and reliable tools for the diagnosis of CPPD disease [9–12]. However, the development of internationally accepted protocols for US imaging in CPPD remains one of the most important research priorities according to a recent survey carried out by the Gout, Hyperuricemia and Crystal-Associated Disease Network (G-CAN) [13]. This research priority has been emphasized in the ACR/EULAR classification criteria for CPPD disease, where both the imaging evidence of CPPD in symptomatic joints and the number of peripheral joints with evidence of CPPD on any imaging modality regardless of symptoms were considered in the criteria, but a clear guidance on which imaging modalities to use and how many joints to image was not given [14]. Obviously, the more peripheral joints are imaged the greater the chance to identify imaging evidence of CPPD [14]. However, an extensive imaging protocol is cost, time and labour intensive and burdens both physicians and patients. On the other hand, a reduced scanning protocol would increase the feasibility and the efficiency of US in clinical practice and in research [15]. Thus, the main aim of this study was to assess the optimal number and sites to be scanned in order to maximize the accuracy of US for diagnosing CPPD disease.

## Methods

### Study population

Consecutive patients aged ≥18 years, with a crystal-proven CPPD disease, who were seen for routine or urgent CPPD care, without applying any further selection criteria, and age- (±2 years) and sex-matched controls with musculoskeletal rheumatic disease without CPP crystals at synovial fluid analysis were prospectively enrolled in this cross-sectional study. Synovial fluid analysis should have been performed no later than 6 months before the enrolment date. Participants were enrolled at the Rheumatology Unit of the Polytechnic University of Marche (Ancona, Italy) and the Department of Rheumatology of the Luigi Sacco University Hospital (Milan, Italy) from January 2021 to November 2022.

Patients with CPPD disease and another coexisting inflammatory arthritis were excluded. Participants were required to not have undergone joint injections in the last 3 months prior to the enrolment date and to not have previous major trauma, fracture or surgery of the joints included in the scanning protocol.

### Clinical assessment

The following clinical and laboratory data were registered in all patients: age, sex, BMI, synovial fluid analysis results and disease duration. In addition, aetiology [i.e. idiopathic, or secondary (familiar or associated with metabolic conditions)] and clinical presentation according to 2011 EULAR recommendations [6] were recorded in patients with CPPD disease.

### Sonographic assessment

Four rheumatologists (E.C., E.F., G.F. and S.S.) carried out the US examinations: E.F. and G.F. had >20 years of experience in the use of musculoskeletal US; E.C. and S.S. had 6 and 5 years of experience in this subject, respectively. All of them were members of the OMERACT US working group — CPPD Task Force. The sonographers were blinded to clinical and laboratory data. Moreover, patients were asked not to share clinical and laboratory information with the sonographers. US examinations were conducted using a My Lab Class C system (Esaote SpA, Genoa, Italy), equipped with two broadband linear transducers (8–13 and 6–18 MHz) and with a Samsung RS85 prestige (Samsung, Seoul, South Korea), equipped with two broadband linear transducers (2–14 and 4–18 MHz).

Each participant underwent a systematic, bilateral and multiplanar US examination of the following joints: shoulders, elbows, wrists, metacarpophalangeal joints, hips, knees, ankles and feet. A total of 22 ‘anatomical targets’ (nine hyaline cartilages, six fibrocartilages, five tendons, one joint capsule and one ligament) were scanned bilaterally in each patient as follows: shoulder (glenoid fibrocartilage, humeral hyaline cartilage and acromioclavicular fibrocartilage), elbow (humeral hyaline cartilage and triceps tendon), wrist (triangular fibrocartilage, dorsal part of the scapho-lunate ligament and volar capsule of the radio-lunate joint), hand (hyaline cartilage of the metacarpophalangeal joints from second to fifth finger), hip (acetabular fibrocartilage and femoral hyaline cartilage), knee (femoral condyles’ hyaline cartilage, meniscal fibrocartilages, patellar and quadriceps tendons), ankle/foot (talar hyaline cartilage, Achilles tendon and plantar fascia). Supplementary Table S1 (available at *Rheumatology* online) shows a detailed description of the scanning protocol based on the 2017 EULAR standardized procedures for US imaging in rheumatology [16].

The grey scale setting parameters were not standardized, and they were manually adapted to enhance the CPP crystal deposit recognition as recommended [17, 18].

CPPD (i.e. within hyaline cartilages, fibrocartilages and tendons/ligaments) was defined according to the Outcome Measure in Rheumatology (OMERACT) definitions (Supplementary Fig. S1 and Supplementary Data S1, available at *Rheumatology* online) [19, 20]. It was scored in a binary fashion at each anatomical target, as the study started before the publication of the OMERACT semiquantitative scoring system [21].

A CPPD joint score ranging from 0 to 20 was calculated as the number of joints included in the scanning protocol with at least one US finding indicative of CPPD. A joint was considered positive if at least one anatomical target in that joint showed US evidence of CPPD.

### Synovial fluid analysis

Synovial fluid aspiration was performed under US guidance as per the standard of care in our departments. In each centre, a biologist, blinded to clinical and imaging data, assessed all the synovial fluid samples using a compensated polarized light microscope. Patients with CPPD disease were classified by the detection of CPP crystals at synovial fluid analysis.

### Data analysis

The accuracy of US in differentiating between patients with CPPD disease and disease controls was investigated using a multi-step approach. As a first step, we investigated which OMERACT US findings were more frequently identified in patients with CPPD disease in comparison with disease controls. Then, we examined the accuracy of each joint (e.g. US findings indicating CPP deposits in the knee) and anatomical target (e.g. US findings indicating CPP deposits in the medial meniscus) in the diagnosis of CPPD disease. Then, we developed and tested the accuracy of reduced US scanning protocols by selecting the most informative joints to be imaged by US using the least absolute shrinkage and selection operator (LASSO) technique [22].

Finally, since the ACR/EULAR criteria took into account CPPD at hyaline cartilages and fibrocartilages only [14], we tested the added value of including CPPD at tendons, capsules and ligaments in our scanning protocol.

### Inter and intra-observer reliability

All the sonographers took part in the latest OMERACT US working group — CPPD subgroup web-based reliability exercise, which yielded substantial to almost perfect agreement in both the web-based and the patient-based exercises [21]. Supplementary Data S2 (available at *Rheumatology* online) reports the inter-observer and the intra-observer reliability of the four sonographers who took part in the present study using the data collected during the OMERACT US working group — CPPD subgroup web-based exercise [21].

### Statistical analysis

Continuous variables were reported as the mean and standard deviation (s.d.) or median and interquartile range (IQR), as appropriate. Categorical variables were presented as absolute frequency and/or corresponding percentage. Continuous variables were compared using Student’s *t-*test or the Mann–Whitney test, as appropriate. Categorical variables were compared using the χ^2^ test.

Starting from the scanning protocol described above, we generated reduced US scanning protocols, including the minimal combination of US findings and anatomical sites to diagnose CPPD, using the LASSO logistic regression [22]. LASSO regression is a method for selecting and fitting covariates that appear in a model and predict the outcome well.

We randomly selected 70% of patients in the cohort as the ‘training set’ and the remaining 30% as the ‘validation set’ balancing the two groups according to age, sex and enrolment sites. The diagnostic accuracy of the scanning protocol was expressed as sensitivity, specificity, positive and negative likelihood ratios with their 95% confidence intervals (95% CIs). The performance of the scanning protocols was compared using the area under the receiver operating characteristic curve (AUROC). In case of a non-significant difference, the shortest scanning protocol (i.e. those with the fewest number of sites) was selected.

Intra-observer and inter-observer reliability were calculated using the kappa coefficient. Intra-observer reliability was assessed with Cohen’s kappa. Inter-observer reliability was evaluated with Light’s kappa. Kappa coefficients were interpreted according to Landis and Koch: κ = 0.00–0.20 means agreement was ‘slight’; κ = 0.21–0.40, ‘fair’; κ = 0.41–0.60, ‘moderate’; κ = 0.61–0.80, ‘substantial’; and κ = 0.81–1.00, ‘almost perfect’ [23].

Sensitivity analyses were carried out to investigate whether the diagnostic accuracy of the reduced scanning protocol was influenced by age (below and above the median age of patients with CPPD), BMI (below and above the median BMI of patients with CPPD), disease duration, the enrolment sites (Ancona and Milan), the experience of the sonographers (master *vs* advanced), and sex in the validation set. Patients with relevant missing data or indeterminate results were excluded from the analyses.

A two-tailed *P*-value < 0.05 was considered significant. Statistical analysis was performed using STATA v.17 (StataCorp, College Station, TX, USA).

### Sample size estimation

Assuming an α error of 5%, an estimation error (*d*) of 8% and a prevalence rate of CPPD disease in our cohort of 50% (enrolment ratio of 1:1), a total of 136 patients (68 CPPD patients and 68 disease controls) would be required to obtain an expected sensitivity of US in the diagnosis of CPPD disease of 91% [24] and an expected specificity of US in the diagnosis of CPPD of 87% [24] in the training set.

### Ethical approval

The study was carried out in accordance with the Declaration of Helsinki, and it was approved by the local Ethics Committee of the Coordinating Centre (Comitato Etico Regione Marche, id CERM: 345/2021). Written informed consent was obtained from all patients before study enrolment. The STAndards for Reporting of Diagnostic accuracy studies 2015 guidelines were adopted [25].

## Results

### Demographic and clinical characteristics of patients with CPPD disease and disease controls

Two hundred and twenty patients were screened, and 204 subjects were enrolled: 102 (50.0%) patients with CPPD and 102 (50.0%) disease controls (Supplementary Fig. S2, available at *Rheumatology* online). Sixteen patients were excluded: 10 patients with CPPD disease and six with knee or hip osteoarthritis. Main reasons for exclusions were knee or hip joint replacement (*n* = 7), a diagnosis of CPPD disease coexisting with another inflammatory arthritis (*n* = 6) and being unwilling to take part in the study (*n* = 3). Table 1 shows demographic, clinical and laboratory findings of the study’s participants. Among disease controls, 30 (29.4%) patients with rheumatoid arthritis, 29 (28.4%) with osteoarthritis (knees, hips or hands), 21 (20.6%) with seronegative spondyloarthropathies, 10 (9.8%) with connective tissue diseases (i.e. systemic lupus erythematosus, systemic sclerosis, Sjögren’s syndrome), 8 (7.8%) with gout, and 4 (3.9%) with polymyalgia rheumatica were enrolled. None of the patients had relevant missing data.

**Table 1.** Demographic and clinical data of patients with CPPD disease and disease controls

	Patients with CPPD disease (*n* = 102)	Disease controls (*n* = 102)	*P*-value
Age, mean (s.d.), years	71.3 (12.0)	71.1 (13.5)	0.51
Sex female, *n* (%)	64 (62.8)	59 (57.8)	0.47
BMI, mean (s.d.), kg/m^2^	25.6 (3.0)	24.7 (4.8)	0.08
Presence of CPP crystal at the SF analysis, *n* (%)	102 (100)	0	<0.01
Duration since the diagnosis, median (IQR)	2 (0.5–6)	3 (1–10)	0.07
CPPD disease aetiology			
Idiopathic, *n* (%)	97 (95.1)	—	—
Associated with predisposing conditions, *n* (%)	5 (4.9)	—	—
EULAR CPPD disease clinical presentation			
Osteoarthritis + CPPD, *n* (%)	40 (39.2)	—	—
Acute CPP crystal arthritis, *n* (%)	53 (52.0)	—	—
Chronic CPP crystal inflammatory arthritis, *n* (%)	9 (8.8)	—	—

CPP: calcium pyrophosphate; CPPD: calcium pyrophosphate deposition; IQR: interquartile range; SF: synovial fluid.

### Prevalence, distribution, and burden of OMERACT US findings indicating CPPD

In the whole scanning protocol, at least one OMERACT US finding indicative of CPPD was detected in 100 out of 102 (98.0%) patients with CPPD disease and in 34 out of 102 (33.3%) disease controls (*P* < 0.01). All the OMERACT US findings were identified in a significantly higher proportion of patients with CPPD disease than disease controls in each anatomical target included in the scanning protocol (all *P* values < 0.01), except for the patellar tendon (*P* = 0.30). Supplementary Table S2 (available at *Rheumatology* online) reports a detailed description of the prevalence and distribution of OMERACT US findings in each anatomical target. Patients with CPPD disease had a significantly higher burden of CPPD (all *P* < 0.01). The median CPPD joint score was 5 (interquartile range: 4–7) whereas it was 0 (interquartile range: 0–1) in disease controls.

### Diagnostic accuracy of OMERACT US findings indicating CPPD

Tables 2 and 3 report the diagnostic accuracy of each anatomical target and each joint, respectively.

**Table 2.** Diagnostic accuracy of US findings indicating CPPD in each anatomic target

Anatomical target	Sensitivity (95% CI), %	Specificity (95% CI), %	Positive likelihood ratio (95% CI)	Negative likelihood ratio (95% CI)
Glenoid fibrocartilage	19.6 (12.4, 28.7)	100 (96.0, 100)	—	0.80 (0.73, 0.88)
Humeral hyaline cartilage	12.8 (7.0, 20.8)	100 (96.0, 100)	—	0.87 (0.81, 0.94)
Acromioclavicular fibrocartilage	57.8 (47.7, 67.6)	90.0 (81.9, 95.3)	5.78 (3.05, 10.99)	0.47 (0.37, 0.59)
Humeral hyaline cartilage	28.4 (19.9, 38.2)	100 (96.0, 100)	—	0.72 (0.63, 0.81)
Triceps tendon	41.2 (31.5, 51.4)	90.0 (81.9, 95.3)	4.12 (2.12, 7.98)	0.65 (0.55, 0.78)
Triangular fibrocartilage	77.5 (68.1, 85.1)	92.2 (84.6, 96.8)	9.96 (4.85, 20.44)	0.24 (0.17, 0.35)
Dorsal component of the SLL	51.0 (40.9, 61.0)	94.4 (87.5, 98.2)	9.18 (3.83, 21.97)	0.52 (0.42, 0.64)
Volar capsule of the radio-lunate joint	38.2 (28.8, 48.4)	93.3 (86.1, 97.5)	5.74 (2.55, 12.91)	0.66 (0.56, 0.78)
Hyaline cartilage of the MCP2	15.7 (9.2, 24.2)	98.9 (94.0, 100)	14.12 (1.91, 104.35)	0.85 (0.78, 0.93)
Hyaline cartilage of the MCP3	15.7 (9.2, 24.2)	100 (96.0, 100)	—	0.84 (0.78, 0.92)
Hyaline cartilage of the MCP4	7.8 (3.5, 14.9)	100 (96.0, 100)	—	0.92 (0.87, 0.98)
Hyaline cartilage of the MCP5	8.8 (4.1, 16.1)	100 (96.0, 100)	—	0.91 (0.86, 0.97)
Acetabular fibrocartilage	53.9 (43.8, 63.8)	97.8 (92.2, 99.7)	24.26 (6.09, 96.66)	0.47 (0.38, 0.58)
Femoral hyaline cartilage	14.7 (8.5, 23.1)	100 (96.0, 100)	—	0.85 (0.79, 0.92)
Femoral condyles’ hyaline cartilage	63.7 (53.6, 73.0)	100 (96.0, 100)	—	0.36 (0.28, 0.47)
Medial meniscus fibrocartilage	89.2 (81.5, 94.5)	95.6 (89.0, 98.8)	20.07 (7.68, 52.44)	0.11 (0.06, 0.20)
Lateral meniscus fibrocartilage	83.3 (74.7, 90.0)	94.4 (87.5, 98.2)	15.0 (6.37, 35.31)	0.18 (0.11, 0.27)
Quadriceps tendon	35.3 (26.1, 45.4)	88.9 (80.5, 94.5)	3.18 (1.67, 6.03)	0.73 (0.62, 0.85)
Patellar tendon	9.8 (4.8, 17.3)	93.3 (86.1, 97.5)	1.47 (0.56, 3.89)	0.97 (0.89, 1.05)
Talar hyaline cartilage	11.8 (6.2, 19.7)	100 (96.0, 100)	—	0.88 (0.82, 0.95)
Achilles tendon	38.2 (28.8, 48.4)	97.8 (92.2, 99.7)	17.21 (4.28, 69.25)	0.63 (0.54, 0.74)
Plantar fascia	14.7 (8.5, 23.1)	96.7 (90.6, 99.3)	4.41 (1.32, 14.75)	0.88 (0.81, 0.96)

CPPD: calcium pyrophosphate deposition; MCP: metacarpophalangeal joint; SLL: scapholunate ligament.

**Table 3.** Diagnostic accuracy of US findings indicating CPPD in each joint

Joint	Number of CPP deposits	Sensitivity (95% CI), %	Specificity (95% CI), %	Positive likelihood ratio (95% CI)	Negative likelihood ratio (95% CI)
Shoulder	≥1	62.8 (52.6, 72.1)	92.2 (85.1, 96.6)	8.0 (4.05, 15.82)	0.40 (0.31, 0.52)
	≥2	28.4 (19.9, 38.2)	98.0 (93.1, 99.8)	14.50 (3.55, 59.18)	0.73 (0.64, 0.83)
Elbow	≥1	65.7 (55.6, 74.8)	91.2 (83.9, 95.9)	7.44 (3.93, 14.11)	0.38 (0.29, 0.50)
	≥2	11.8 (6.2, 19.7)	98.0 (93.1, 99.8)	6.0 (1.38, 26.14)	0.90 (0.83, 0.97)
Wrist	≥1	80.4 (71.4, 87.6)	89.2 (81.5, 94.5)	7.45 (4.23, 13.13)	0.22 (0.15, 0.33)
	≥2	55.9 (45.7, 65.7)	98.0 (93.1, 99.8)	28.50 (7.15, 113.63)	0.45 (0.36, 0.56)
Hand	≥1	23.5 (15.7, 33.0)	99.0 (94.7, 99.9)	24.0 (3.31, 174.09)	0.77 (0.69, 0.86)
	≥2	9.8 (4.8, 17.3)	99.9 (96.5, 99.9)	—	0.90 (0.85, 0.96)
Hip	≥1	55.9 (45.7, 65.7)	98.0 (93.1, 99.8)	28.50 (7.15, 113.63)	0.45 (0.36, 0.56)
	≥2	12.8 (7.0, 20.8)	99.9 (96.5, 99.9)	—	0.87 (0.81, 0.94)
Knee	≥1	97.1 (91.6, 99.4)	82.4 (73.6, 89.2)	5.50 (3.61, 8.38)	0.04 (0.01, 0.11)
	≥2	85.3 (76.9, 91.5)	96.1 (90.3, 98.9)	21.75 (8.29, 57.03)	0.15 (0.10, 0.24)
Ankle/foot	≥1	53.9 (43.8, 63.8)	95.1 (88.9, 98.4)	11.0 (4.59, 26.35)	0.48 (0.39, 0.60)
	≥2	17.7 (10.8, 26.5)	99.9 (96.5, 99.9)	—	0.82 (0.75, 0.90)

CPP: calcium pyrophosphate, CPPD: calcium pyrophosphate deposition, US: ultrasonography.

### Development and testing of an US scanning protocol for the diagnosis of CPPD disease

One hundred and forty-three (72 patients with CPPD disease and 71 disease controls) and 61 (30 patients with CPPD disease and 31 disease controls) individuals were randomly assigned to the ‘training set’ and the ‘validation set’, respectively.

According to the LASSO technique, the knee (standardized LASSO coefficient: 6.66), the wrist (standardized LASSO coefficient: 3.49) and the hip (standardized LASSO coefficient: 2.20) were the most informative joints to be imaged by US followed by the metacarpophalangeal joints (standardized LASSO coefficient: 0.80), the shoulder (standardized LASSO coefficient: 0.62), the ankle (standardized LASSO coefficient: −0.24) and the elbow (standardized LASSO coefficient: 0.05) in the training set. Then, the LASSO technique was applied to each joint to identify the most informative anatomical targets to be imaged in each joint (Table 4).

**Table 4.** Identification of the most informative anatomical targets in each joint

Joint	Standardized LASSO coefficient	Anatomical target	Standardized LASSO coefficient
Shoulder	0.62	Glenoid fibrocartilage	2.57
		Humeral hyaline cartilage	2.11
		Acromioclavicular fibrocartilage	1.08
Elbow	0.05	Humeral hyaline cartilage	3.64
		Triceps tendon	0.98
Wrist	3.49	Triangular fibrocartilage	1.67
		Volar capsule of the radio-lunate joint	1.64
		Dorsal component of the scapho-lunate ligament	0.87
MCP	0.80	Hyaline cartilage of the 3rd metacarpophalangeal joint	0.59
		Hyaline cartilage of the 2nd metacarpophalangeal joint	0.40
		Hyaline cartilage of the 4th metacarpophalangeal joint	0.01
		Hyaline cartilage of the 5th metacarpophalangeal joint	0.01
Hip	2.20	Acetabular fibrocartilage	1.53
		Femoral hyaline cartilage	1.34
Knee	6.66	Femoral condyles’ hyaline cartilage	4.97
		Medial meniscus fibrocartilage	2.09
		Lateral meniscus fibrocartilage	0.98
		Quadriceps tendon	0.82
		Patellar tendon	−1.63
Ankle/foot	0.24	Talar hyaline cartilage	1.17
		Achilles tendon	1.04
		Plantar fascia	0.21

LASSO: least absolute shrinkage and selection operator technique; MCP: metacarpophalangeal joints.

**Figure 1.**
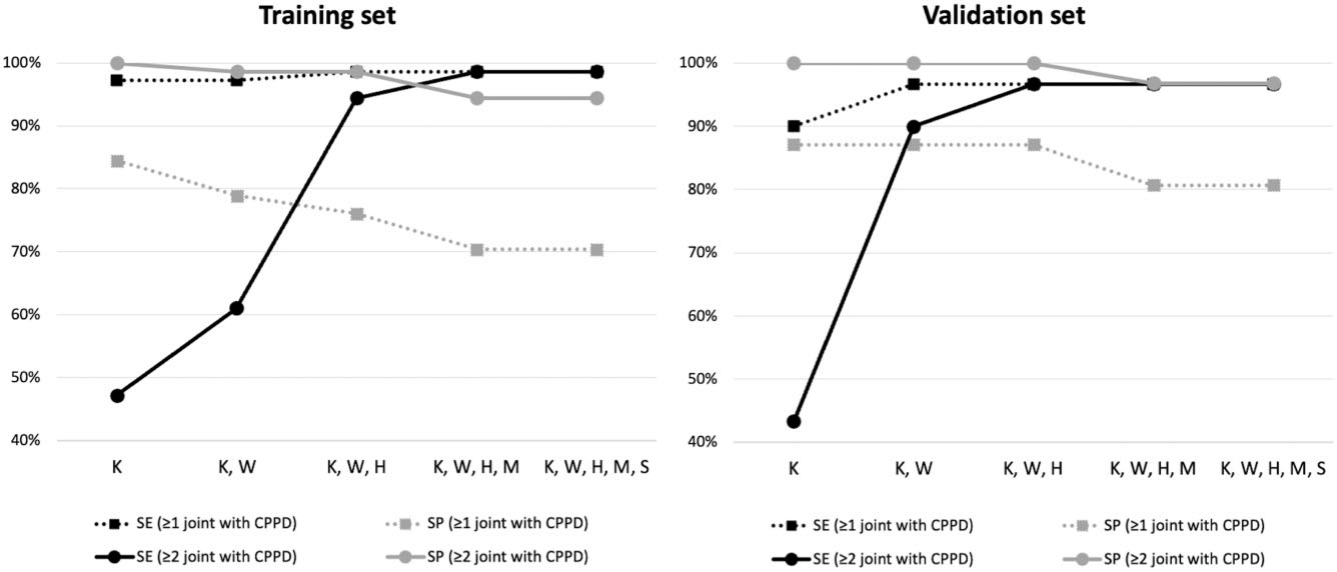
Variations in US accuracy for the diagnosis of CPPD using different scanning protocols and cut-off values in the training and validation sets. Solid line: two or more joints with CPPD are required to diagnose patients as having CPPD disease; dotted line: one or more joints with CPPD are required to diagnose patients as having CPPD disease. Refer to Supplementary Tables S3 and S4 for further details (available at *Rheumatology* online). CPPD: calcium pyrophosphate deposition; H: hips; K: knees; M: metacarpophalangeal joints; S: shoulders; SE: sensitivity; SP: specificity; W: wrists

Supplementary Tables S3 and S4 (available at *Rheumatology* online) report the performance of different US scanning protocols developed with the LASSO technique in the training and validation sets. Figure 1 shows the variation in the US accuracy for the diagnosis of CPPD using different scanning protocols and cut-off values in the training and validation sets. Supplementary Fig. 3 (available at *Rheumatology* online) and Table 5 display the selected US scanning protocol for the diagnosis of CPPD.

**Table 5.** Accuracy of the proposed US scanning protocol for the diagnosis of CPPD disease

Number of joints with at least one US finding indicative of CPPD	Sensitivity (95% CI), %	Specificity (95% CI), %	Positive likelihood ratio (95% CI)	Negative likelihood ratio (95% CI)
Training set				
≥1	98.6 (92.5, 99.9)	76.1 (64.5, 85.4)	4.1 (2.7, 6.2)	0.02 (0.00, 0.13)
** ≥2**	**94.4 (86.4, 98.5)**	**98.6 (92.5, 99.9)**	**67.1 (9.6, 469.9)**	**0.06 (0.02, 0.15)**
≥3	81.9 (71.1, 90.0)	100.0 (94.9, 100)	—	0.18 (0.11, 0.30)
Validation set				
≥1	96.7 (82.8, 99.9)	87.1 (70.2, 6.4)	7.5 (3.0, 18.7)	0.04 (0.01, 0.26)
** ≥2**	**96.7 (82.8, 99.9)**	**100.0 (88.8, 100)**	—	**0.03 (0.00, 0.23)**

The scanning protocol includes the bilateral assessment of the wrist (the triangular fibrocartilage), the hip (the acetabular fibrocartilage and the femoral hyaline cartilage) and the knee (the femoral condyles’ hyaline cartilage, the medial meniscus fibrocartilage and the lateral meniscus fibrocartilage). The most accurate cut-off value to discriminate cases and controls is highlighted in bold. CPPD: calcium pyrophosphate deposition.

The accuracy of the proposed US scanning protocol was not significantly improved by the inclusion of non-cartilaginous structures (i.e. tendons, joint capsules and ligaments) (*P* = 0.71 in the training set and *P* = 0.63 in the validation set).

### Sensitivity analyses

The diagnostic accuracy of the six-joint scanning protocol was not influenced by any of the following covariates: age (*P* = 0.92), BMI (*P* = 0.32), disease duration (*P* = 0.63), enrolment site (*P* = 0.12), sonographers’ level of experience (*P* = 0.23) and sex (*P* = 0.51).

### Feasibility of the selected US scanning protocol

The feasibility of this six-joint scanning protocol was tested after the development of the scanning protocol in a group of 20 consecutive patients with joint pain and without a definite diagnosis. The mean (s.d.) time required to complete the six-joint scanning protocol was 12.5 (5.3) min, while the average time for the 20-joint extended protocol was 28 (6.7) min (*P* < 0.01).

## Discussion

The main aim of the present study was to investigate the core set of joints that ought to be scanned by US for the diagnosis of CPPD disease balancing feasibility with diagnostic accuracy.

In this study, we used a 20-joint US scanning protocol, and, to the best of our knowledge, it is the most comprehensive US assessment published so far in CPPD disease. However, the use of such an extended scanning protocol has some drawbacks: it requires extensive knowledge of musculoskeletal sono-anatomy, and it is time-consuming and not feasible in clinical practice. Therefore, we evaluated which US findings and which joints are more informative to be scanned to diagnose patients with CPPD disease, using a LASSO logistic regression. This technique is an automatic method for selecting the most informative variables that does not rely on arbitrary thresholds. In the present study, knees, wrists and hips were the most informative joints to be imaged by US followed by metacarpophalangeal joints, shoulders, ankles and elbows.

The paramount importance of US assessment of knees and wrists in the diagnosis of CPPD disease is supported by previous US studies, which were mainly focused on these joints [12]. Indeed, in a recent systematic literature review exploring the prevalence of CPPD by different imaging techniques [12], the knee was evaluated in 15 (68.2%) of 22 US studies, the wrist in 8 (36.4%), the ankle/foot in 7 (31.8%), and the hip, the shoulder and the hand in 2 (9.1%). On the other hand, our results indicate that the hip should be included in a scanning protocol evaluating the presence of CPPD, as it is a common target of CPPD. Moreover, its importance has been emphasized in previous studies [26–28].

Identifying CPPD at least one site at the knee level had a sensitivity >90% for the diagnosis of CPPD disease. Adding other joints (i.e. wrists and hips) led to a small increase in sensitivity, which did not increase further with the addition of other joints (e.g. metacarpophalangeal joints and shoulders). However, the specificity of this cut-off value (i.e. ≥1 joints with CPPD) was suboptimal (<90%), and it decreased as the number of joints increased. As reported in Fig. 1, the detection of CPPD in ≥2 joints in a reduced six-joint US scanning protocol that included knees, wrists and hips was the best trade-off between sensitivity and specificity, which were >90%. This approach may increase the feasibility of US in the diagnostic work-up of patients with CPPD disease without any significant loss in accuracy.

Although US examination of knees, wrists and hips is of the utmost importance in the US diagnosis of CPPD disease, the evaluation of other joints such as shoulders, ankles, MCPs and elbows may be required in specific circumstances (e.g. one of these joints is clinically involved or was involved in the past [24], <2 joints with CPPD in the reduced US scanning protocol, or when knees/hips are not assessable due to joint replacement) or to rule out other diseases.

In this cross-sectional study, we found that 75% of patients with CPPD disease and none of disease controls had four or more joints with CPPD in an extended scanning protocol of 20 joints. This finding confirms that there is a systemic predisposition to CPPD [27, 29, 30] and, also, the importance of CPPD burden in the diagnosis of CPPD disease, as highlighted in the 2023 ACR/EULAR classification criteria [14]. Therefore, our findings may be useful not only in clinical practice, but also in research when deciding a set of joints to be imaged to classify patients as having CPPD disease.

The results of the present paper confirm the higher importance of cartilaginous structures (i.e. hyaline cartilages and fibrocartilages) in comparison with non-cartilaginous tissues (i.e. tendons, ligaments and joint capsules) in the diagnosis of patients with CPPD disease. This aspect has already been acknowledged by the experts involved in the development of the ACR/EULAR classification criteria [14]. However, to date, it was mainly based on expert opinion. Our data support the approach taken by ACR/EULAR CPPD classification criteria [14]. Indeed, fibrocartilages and hyaline cartilages were more informative for scanning than tendons, ligaments and joint capsules in all joints included in the extended scanning protocol. Also, the inclusion of non-cartilaginous structures did not significantly improve the diagnostic accuracy of the reduced scanning protocol. In addition, OMERACT US definitions for CPPD within hyaline cartilages and fibrocartilages were found to be more reliable than those for CPPD within tendons and synovial fluid in OMERACT reliability exercises [19, 20].

The present study has several strengths. First, US assessments were performed by four sonographers from two Italian Centres using standardized US scanning protocol and US definitions for CPPD. Moreover, their intra- and inter-observer reliability on static images was almost perfect. Second, data were divided into training and validation sets to avoid overfitting and to increase the external validity of our results. Third, patients with CPPD disease and disease controls were systematically identified using synovial fluid analysis as the current reference standard.

We must also acknowledge some limitations. First, the OMERACT US definitions for CPPD have been validated against histology at knees only [11]. Similarly, the intra- and inter-observer reliability of the OMERACT US definitions for CPPD has been found to be acceptable at knees and wrists only [19]. Therefore, further studies are needed to confirm the external validity and the reliability of OMERACT US definitions for CPPD at other joints such as the hip. Second, US findings indicative of CPPD were scored as presence/absence. Therefore, despite the great experience of the sonographers, we could not exclude that other mimickers (i.e. primary degenerative calcifications and scar tissue) may have biased our results. Third, patients’ symptoms were not considered in this cross-sectional study. Therefore, our scanning protocol did not take into account the involvement of specific joints in the personal medical history. Knees, wrists and hips should be considered as the most informative joints to be scanned by US. However, other joints (e.g. those clinically involved in the present or in the past) may be imaged as they may provide important diagnostic clues [14, 24]. Fourth, although CPPD is a systemic disease, we found a relatively low prevalence of bilateral CPPD. According to a recent systematic literature review carried out by the OMERACT CPPD working group [12], no US data regarding the bilateral involvement of CPPD are available in the literature. Future studies are required to clarify this point.

In conclusion, a reduced six-joint US scanning protocol that included hyaline cartilages and fibrocartilages of knees, wrists and hips showed excellent accuracy and good feasibility for the diagnosis of CPPD disease. Knees, wrists and hips should always be included in US scanning protocols for CPPD disease, whereas the evaluation of other joints may be required in specific circumstances.

## Supplementary Material

kead565_Supplementary_Data

## Data Availability

The study protocol and the data that support the findings of this study are available from the corresponding author upon reasonable request.
