# Supplementary material for: Diagnosis of calcium pyrophosphate crystal deposition disease by ultrasonography: how many and which sites should be scanned?
Source: Rheumatology (Oxford). 2023 Oct 26;63(8):2205–12. doi: 10.1093/rheumatology/kead565 (PMC11292044; doi:10.1093/rheumatology/kead565)

**APPENDIX**

**Supplementary Table S1.** US scanning protocol page 2

**Supplementary Table S2.** Prevalence and distribution of CPPD in patients with CPPD disease and disease controls page 3

**Supplementary Table S3.** Accuracy of US scanning protocols developed with the LASSO technique in the training set for the diagnosis of CPPD disease page 4

**Supplementary Table S4.** Accuracy of US scanning protocols developed with the LASSO technique in the validation set for the diagnosis of CPPD disease page 5

**Supplementary Data S1**. US appearance of CPPD in different tissues page 6

**Supplementary Data S2**. Intra-reader and inter-reader reliability of the sonographers who took part into the study page 7

**Supplementary Figure S1**. Representative pictures showing CPPD in different anatomical targets page 8

**Supplementary Figure S2**. Flow chart of the study’s participants page 9

**Supplementary Figure S3.** Proposed US scanning protocol for the diagnosis of CPPD disease. page 10

| **Supplementary Table S1**. US scanning protocol | | |
| --- | --- | --- |
| **Joint** | **Anatomical target** | **EULAR Standard Scan** [16] |
| Shoulder | Glenoid fibrocartilage | S21 |
|  | Humeral hyaline cartilage | S21, S22 |
|  | Acromioclavicular fibrocartilage | S05 |
| Elbow | Humeral hyaline cartilage | E01, E02 |
|  | Triceps tendon | E15, E16 |
| Wrist | Triangular fibrocartilage | W10, W11 |
|  | Dorsal component of the scapho-lunate ligament | W07, W08 |
|  | Volar capsule of the radio-lunate joint | W29, W39, W40 |
| Metacarpophalangeal joint | Hyaline cartilage of the metacarpophalangeal joints from 2^nd^ to 5^th^ digit | W19 |
| Hip | Acetabular fibrocartilage | H1, H2, H4 |
|  | Femoral hyaline cartilage | H1, H2, H3 |
| Knee | Femoral condyles’ hyaline cartilage | K20, K21 |
|  | Medial meniscus fibrocartilage | K06 |
|  | Lateral meniscus fibrocartilage | K08 |
|  | Quadriceps tendon | K2, K3, K5 |
|  | Patellar tendon | K11, K12, K14, K16 |
| Ankle/foot | Talar hyaline cartilage | A02 |
|  | Achilles tendon | A47, A49, A51 |
|  | Plantar fascia | A52 |

| **Supplementary Table S2**. Prevalence and distribution of CPPD in patients with CPPD disease and disease controls | | | | | |
| --- | --- | --- | --- | --- | --- |
| **Joint** | **Anatomic target** | **Patients with CPPD disease (n=102)** | | **Disease controls (n=102)** | |
|  |  | **At least one n (%)** | **Bilateral n (%)** | **At least one n (%)** | **Bilateral n (%)** |
| Shoulder | Glenoid fibrocartilage and/or humeral hyaline cartilage and/or acromioclavicular fibrocartilage | 64 (62.7%) | 29 (28.4%) | 8 (7.8%) | 2 (2.0%) |
|  | Glenoid fibrocartilage | 20 (19.6%) | 10 (9.8%) | 0 | 0 |
|  | Humeral hyaline cartilage | 13 (12.8%) | 4 (3.9%) | 0 | 0 |
|  | Acromioclavicular fibrocartilage | 59 (57.8%) | 29 (28.4%) | 9 (8.8%) | 2 (2.0%) |
| Elbow | Humeral hyaline cartilage and/or triceps tendon | 67 (65.7%) | 12 (11.8%) | 9 (8.8%) | 2 (2.0%) |
|  | Humeral hyaline cartilage | 29 (28.4%) | 6 (5.9%) | 0 | 0 |
|  | Triceps tendon | 42 (41.2%) | 10 (9.8%) | 9 (8.8%) | 2 (2.0%) |
| Wrist | Triangular fibrocartilage and/or dorsal component of the SLL and/or volar capsule of the radio-lunate joint | 82 (80.4%) | 57 (55.9%) | 11 (10.8%) | 2 (2.0%) |
|  | Triangular fibrocartilage | 79 (77.5%) | 41 (40.2%) | 7 (6.9%) | 1 (1.0%) |
|  | Dorsal component of the SLL | 52 (51.0%) | 20 (19.6%) | 5 (4.9%) | 1 (1.0%) |
|  | Volar capsule of the radio-lunate joint | 39 (38.2%) | 17 (16.7%) | 6 (5.9%) | 0 |
| Hand | Hyaline cartilage of the MCP2-5 | 24 (23.5%) | 10 (9.8%) | 1 (1.0%) | 0 |
|  | Hyaline cartilage of the MCP2 | 16 (15.7%) | 4 (3.9%) | 1 (1.0%) | 0 |
|  | Hyaline cartilage of the MCP3 | 16 (15.7%) | 4 (3.9%) | 0 | 0 |
|  | Hyaline cartilage of the MCP4 | 8 (7.8%) | 2 (2.0%) | 0 | 0 |
|  | Hyaline cartilage of the MCP5 | 9 (8.8%) | 1 (1.0%) | 0 | 0 |
| Hip | Acetabular fibrocartilage and/or femoral hyaline cartilage | 57 (55.9%) | 13 (12.7%) | 2 (2.0%) | 0 |
|  | Acetabular fibrocartilage | 55 (53.9%) | 13 (12.7%) | 2 (2.0%) | 0 |
|  | Femoral hyaline cartilage | 15 (14.7%) | 5 (4.9%) | 0 | 0 |
| Knee | Femoral condyles’ hyaline cartilage and/or medial meniscus fibrocartilage and/or lateral meniscus fibrocartilage and/or quadriceps tendon | 99 (97.1%) | 67 (65.7%) | 18 (17.7%) | 4 (3.9%) |
|  | Femoral condyles’ hyaline cartilage | 65 (63.7%) | 17 (16.7%) | 0 | 0 |
|  | Medial meniscus fibrocartilage | 91 (89.2%) | 51 (50.0%) | 4 (3.9%) | 1 (1.0%) |
|  | Lateral meniscus fibrocartilage | 85 (83.3%) | 49 (48.0%) | 5 (4.9%) | 1 (1.0%) |
|  | Quadriceps tendon | 36 (35.3%) | 20 (19.6%) | 10 (10.8%)) | 1 (1.0%) |
|  | Patellar tendon | 10 (9.8%) | 7 (6.9%) | 6 (5.9%) | 1 (1.0%) |
| Ankle/foot | Talar hyaline cartilage and/or Achilles tendon and/or plantar fascia | 47 (46.1%) | 18 (17.6%) | 5 (4.9%) | 2 (2.0%) |
|  | Talar hyaline cartilage | 12 (11.8%) | 8 (7.8%) | 0 | 0 |
|  | Achilles tendon | 39 (38.2%) | 14 (13.7%) | 2 (2.0%) | 1 (1.0%) |
|  | Plantar fascia | 15 (14.7%) | 4 (3.9%) | 3 (2.9%) | 1 (1.0%) |
| **CPPD**: calcium pyrophosphate deposition, **MCP**: metacarpophalangeal joint, **SLL**: scapholunate ligament. | | | | | |

| **Supplementary Table S3.** Accuracy of US scanning protocols developed with the LASSO technique in the training set for the diagnosis of CPPD disease | | | | |
| --- | --- | --- | --- | --- |
|  | **Sensitivity (%) (95%CI)** | **Specificity (%) (95%CI)** | **Positive likelihood ratio (95%CI)** | **Negative likelihood ratio (95%CI)** |
| **Knees, wrists, hips, MCPs, shoulders, ankles, elbows** (AUROC: 0.99 (95%CI: 0.97-1.0)) | | | | |
| ≥1 joint with CPPD | 98.6 (92.5-99.9) | 70.4 (58.4-80.7) | 3.3 (2.3-4.8) | 0.02 (0.00-0.14) |
| ≥2 joint with CPPD | 98.6 (92.5-99.9) | 94.4 (86.2-98.4) | 17.5 (6.8-45.4) | 0.01 (0.00-0.10) |
| ≥3 joint with CPPD | 98.6 (92.5-99.9) | 98.6 (92.4-99.9) | 70.0 (10.0-490.3) | 0.01 (0.00-0.10) |
| ≥4 joint with CPPD | 76.4 (64.9-85.6) | 100.0 (94.9-100) | - | 0.24 (0.16-0.36) |
| **Knees, wrists, hips, MCPs, shoulders, ankles** (AUROC: 0.99 (95%CI: 0.97-1.0)) | | | | |
| ≥1 joint with CPPD | 98.6 (92.5-99.9) | 70.4 (58.4-80.7) | 3.3 (2.3-4.8) | 0.02 (0.00-0.14) |
| ≥2 joint with CPPD | 98.6 (92.5-99.9) | 94.4 (86.2-98.4) | 17.5 (6.8-45.4) | 0.01 (0.00-0.10) |
| ≥3 joint with CPPD | 97.2 (90.3-99.7) | 98.6 (92.4-99.9) | 69.0 (9.9-483.5) | 0.03 (0.01-0.11) |
| ≥4 joint with CPPD | 75.0 (63.4-84.5) | 100.0 (94.9-100) | - | 0.25 (0.17-0.37) |
| **Knees, wrists, hips, MCPs, shoulders** (AUROC: 0.99 (95%CI: 0.97-1.0)) | | | | |
| ≥1 joint with CPPD | 98.6 (92.5-99.9) | 70.4 (58.4-80.7) | 3.3 (2.3-4.8) | 0.02 (0.00-0.14) |
| ≥2 joint with CPPD | 98.6 (92.5-99.9) | 94.4 (86.2-98.4) | 17.5 (6.8-45.4) | 0.01 (0.00-0.10) |
| ≥3 joint with CPPD | 94.4 (86.4-98.5) | 100.0 (94.9-100) | - | 0.06 (0.02-0.14) |
| **Knees, wrists, hips, MCPs** (AUROC: 0.99 (95%CI: 0.97-1.0)) | | | | |
| ≥1 joint with CPPD | 98.6 (92.5-99.9) | 70.4 (58.4-80.7) | 3.3 (2.3-4.8) | 0.02 (0.00-0.14) |
| ≥2 joint with CPPD | 98.6 (92.5-99.9) | 94.4 (86.2-98.4) | 17.5 (6.8-45.4) | 0.01 (0.00-0.10) |
| ≥3 joint with CPPD | 88.9 (79.2-95.1) | 100.0 (94.9-100) | - | 0.11 (0.06-0.21) |
| **Knees, wrists, hips** (AUROC: 0.99 (95%CI: 0.97-1.0)) | | | | |
| ≥1 joint with CPPD | 98.6 (92.5-99.9) | 76.1 (64.5-85.4) | 4.1 (2.7-6.2) | 0.02 (0.00-0.13) |
| ≥2 joint with CPPD | 94.4 (86.4-98.5) | 98.6 (92.5-99.9) | 67.1 (9.6-469.9) | 0.06 (0.02-0.15) |
| ≥3 joint with CPPD | 81.9 (71.1-90.0) | 100.0 (94.9-100) | - | 0.18 (0.11-0.30) |
| **Knees, wrists** (AUROC: 0.98 (95%CI: 0.95-1.0)) | | | | |
| ≥1 joint with CPPD | 97.2 (90.3-99.7) | 78.9 (67.6-87.7) | 4.6 (2.9-7.2) | 0.04 (0.01-0.14) |
| ≥2 joint with CPPD | 93.1 (84.5-97.7) | 98.6 (92.4-99.9) | 66.1 (9.4-463.1) | 0.07 (0.03-0.16) |
| ≥3 joint with CPPD | 61.1 (48.9-72.4) | 100.0 (94.9-100) | - | 0.39 (0.29-0.52) |
| **Knees** (AUROC: 0.95 (95%CI: 0.91-0.98)) | | | | |
| ≥1 joint with CPPD | 97.2 (90.3-99.7) | 84.5 (74.0-92.0) | 6.3 (3.6-10.8) | 0.03 (0.01-0.13) |
| ≥2 joint with CPPD | 47.2 (35.3-59.4) | 100.0 (94.9-100) | - | 0.53 (0.42-0.66) |
| **ACR**: American College of Rheumatology, **AUROC**: area under the receiver operating characteristic, **CPPD**: calcium pyrophosphate deposition, **EULAR**: European Alliance of Associations for Rheumatology, **LASSO**: least absolute shrinkage and selection operator, **MCP**: metacarpophalangeal joint. | | | | |
|  | | | | |

| **Supplementary Table S4.** Accuracy of US scanning protocols developed with the LASSO technique in the validation set for the diagnosis of CPPD disease | | | | |
| --- | --- | --- | --- | --- |
|  | **Sensitivity (%) (95%CI)** | **Specificity (%) (95%CI)** | **Positive likelihood ratio (95%CI)** | **Negative likelihood ratio (95%CI)** |
| **Knees, wrists, hips, MCPs, shoulders, ankles, elbows** (AUROC: 0.98 (95%CI: 0.94-1.0)) | | | | |
| ≥1 joint with CPPD | 96.7 (82.8-99.9) | 80.7 (62.5-92.6) | 5.0 (2.4-10.3) | 0.04 (0.01-0.29) |
| ≥2 joints with CPPD | 96.7 (82.8-99.9) | 96.8 (83.3-99.9) | 30.0 (4.4-206.3) | 0.03 (0.01-0.24) |
| ≥3 joints with CPPD | 90.0 (73.5-97.9) | 100.0 (88.8-100) | - | 0.10 (0.03-0.29) |
| **Knees, wrists, hips, MCPs, shoulders, ankles** (AUROC: 0.98 (95%CI: 0.94-1.0)) | | | | |
| ≥1 joint with CPPD | 96.7 (82.8-99.9) | 80.7 (62.5-92.6) | 5.0 (2.4-10.3) | 0.04 (0.01-0.29) |
| ≥2 joints with CPPD | 96.7 (82.8-99.9) | 96.8 (83.3-99.9) | 30.0 (4.4-206.3) | 0.03 (0.01-0.24) |
| ≥3 joints with CPPD | 90.0 (73.5-97.9) | 100.0 (88.8-100) | - | 0.10 (0.03-0.29) |
| **Knees, wrists, hips, MCPs, shoulders** (AUROC: 0.98 (95%CI: 0.94-1.0)) | | | | |
| ≥1 joint with CPPD | 96.7 (82.8-99.9) | 80.7 (62.5-92.6) | 5.0 (2.4-10.3) | 0.04 (0.01-0.29) |
| ≥2 joints with CPPD | 96.7 (82.8-99.9) | 96.8 (83.3-99.9) | 30.0 (4.4-206.3) | 0.03 (0.01-0.24) |
| ≥3 joints with CPPD | 90.0 (73.5-97.9) | 100.0 (88.8-100) | - | 0.10 (0.03-0.29) |
| **Knees, wrists, hips, MCPs** (AUROC: 0.98 (95%CI: 0.94-1.0)) | | | | |
| ≥1 joint with CPPD | 96.7 (82.8-99.9) | 80.7 (62.5-92.6) | 5.0 (2.4-10.3) | 0.04 (0.01-0.29) |
| ≥2 joints with CPPD | 96.7 (82.8-99.9) | 96.8 (83.3-99.9) | 30.0 (4.4-206.3) | 0.03 (0.01-0.24) |
| ≥3 joints with CPPD | 90.0 (73.5-97.9) | 100.0 (88.8-100) | - | 0.10 (0.03-0.29) |
| **Knees, wrists, hips** (AUROC: 0.98 (95%CI: 0.94-1.0)) | | | | |
| ≥1 joint with CPPD | 96.7 (82.8-99.9) | 87.1 (70.2-6.4) | 7.5 (3.0-18.7) | 0.04 (0.01-0.26) |
| ≥2 joints with CPPD | 96.7 (82.8-99.9) | 100.0 (88.8-100) | - | 0.03 (0.00-0.23) |
| **Knees, wrists** (AUROC: 0.98 (95%CI: 0.94-1.0)) | | | | |
| ≥1 joint with CPPD | 96.7 (82.8-99.9) | 87.1 (70.2-6.4) | 7.5 (3.0-18.7) | 0.04 (0.01-0.26) |
| ≥2 joints with CPPD | 90.0 (73.5-97.9) | 100.0 (88.8-100) | - | 0.10 (0.03-0.29) |
| **Knees** (AUROC: 0.97 (95%CI: 0.94-1.0)) | | | | |
| ≥1 joint with CPPD | 90.0 (73.5-97.9) | 87.1 (70.2-6.4) | 7.0 (2.8-17.5) | 0.11 (0.04-0.34) |
| ≥2 joints with CPPD | 43.3 (25.5-62.6) | 100.0 (88.8-100) | - | 0.60 (0.41-0.77) |
| **ACR**: American College of Rheumatology, **AUROC**: area under the receiver operating characteristic, **CPPD**: calcium pyrophosphate deposition, **EULAR**: European Alliance of Associations for Rheumatology, **LASSO**: least absolute shrinkage and selection operator, **MCP**: metacarpophalangeal joint. | | | | |
|  | | | | |

**Supplementary Data S1**. US appearance of CPPD in different tissues [19,19]

**Fibrocartilage CPPD:** Hyperechoic (similar to the bone cortex echogenicity) deposits of variable shape, localised within the fibrocartilage that remain fixed and move together with the fibrocartilage during dynamic assessment. They do not create posterior shadowing.

**Hyaline cartilage CPPD:** Hyperechoic (similar to the bone cortex echogenicity) deposits of variable shape and size, localised within the hyaline cartilage that remain fixed and move together with the hyaline cartilage during dynamic assessment. They do not create posterior shadowing.

**Tendon and ligament CPPD:** Hyperechoic (similar to the bone cortex echogenicity and in relation to tendon echogenicity) deposits, localised within the tendon/ligament, that are not affected by anisotropy as the surrounding tissue and do not create posterior shadowing. Usually, they have a linear shape parallel to the tendon/ligament fibrillar structure that are not in continuity with bony endings. They remain fixed and move together with the tendon/ligament during dynamic assessment.

**Supplementary Data S2**. Intra-reader and inter-reader reliability of the sonographers who took part into the study

The reliability was calculated using the data collected during the web based OMERACT US CPPD exercise [21]. and a binary score (where OMERACT grades 1, 2 and 3 were considered as indicative of CPPD deposits). The intra-reader reliability was almost perfect using the OMERACT semiquantitative score for CPPD (unweighted kappa: 0.96 (95%CI: 0.93-0.98)) and a binary score for CPPD (unweighted kappa: 0.99 (95%CI: 0.98-1.0). Similar results were observed for the inter-reader reliability using the OMERACT semiquantitative score for CPPD (unweighted kappa: 0.87 (95%CI: 0.84-0.91)) and a binary score for CPPD (unweighted kappa: 0.98 (95%CI: 0.96-1.0).

**Supplementary Figure S1**. Representative pictures showing CPPD in different anatomical targets


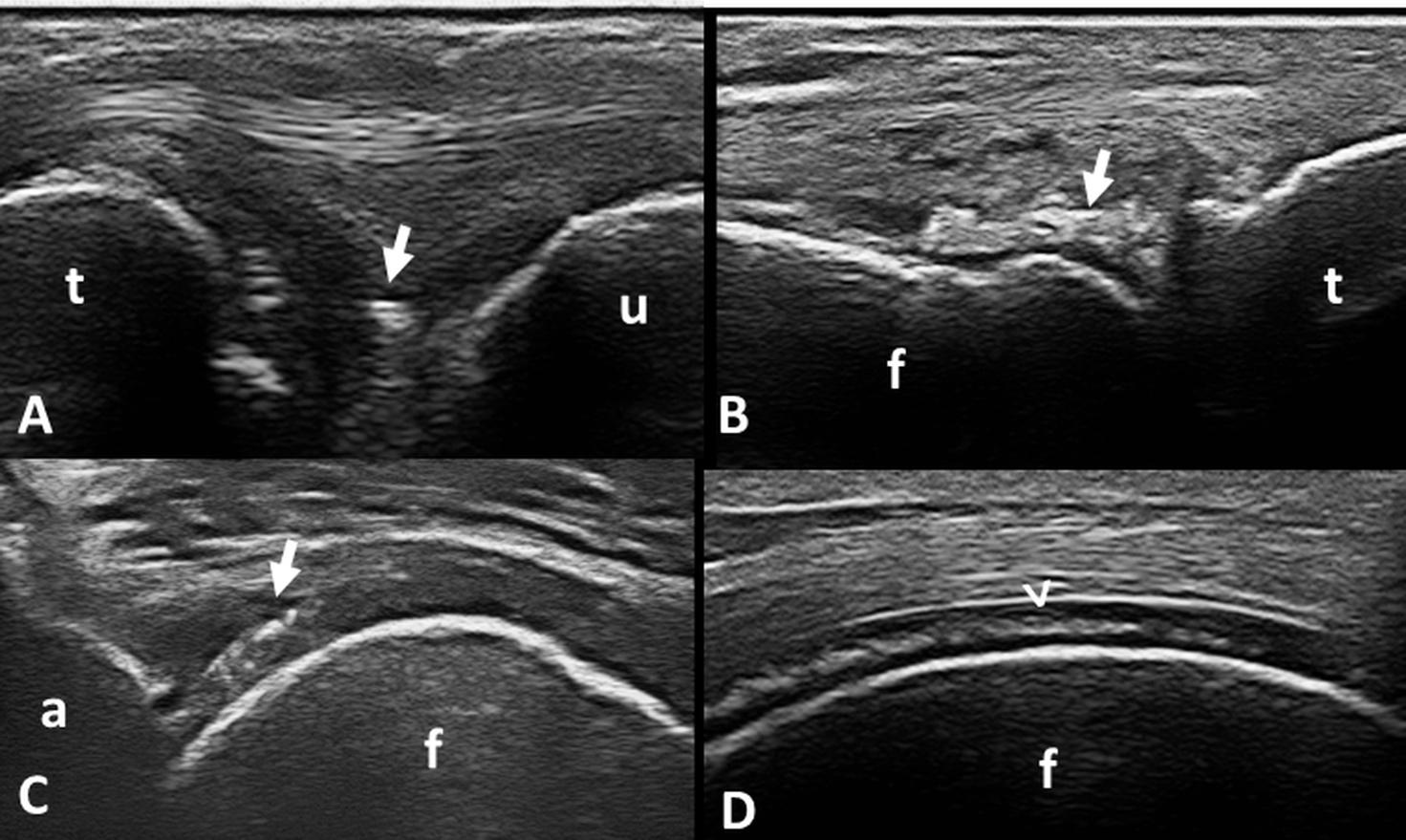


A: Wrist, longitudinal scan of the triangular fibrocartilage complex, t: triquetrum, u: ulna.

B: Knee, longitudinal scan of the lateral meniscus, f: femur, t: tibia.

C: Hip, longitudinal scan of the acetabular fibrocartilage, a: acetabulum, f: femur.

D: Knee, longitudinal scan of the medial femoral condyle’s hyaline cartilage, f: femur.

**Arrows**: CPP deposits at fibrocartilages, **arrowhead**: CPP deposits at hyaline cartilage level.

**Supplementary Figure S2**. Flow chart of the study’s participants


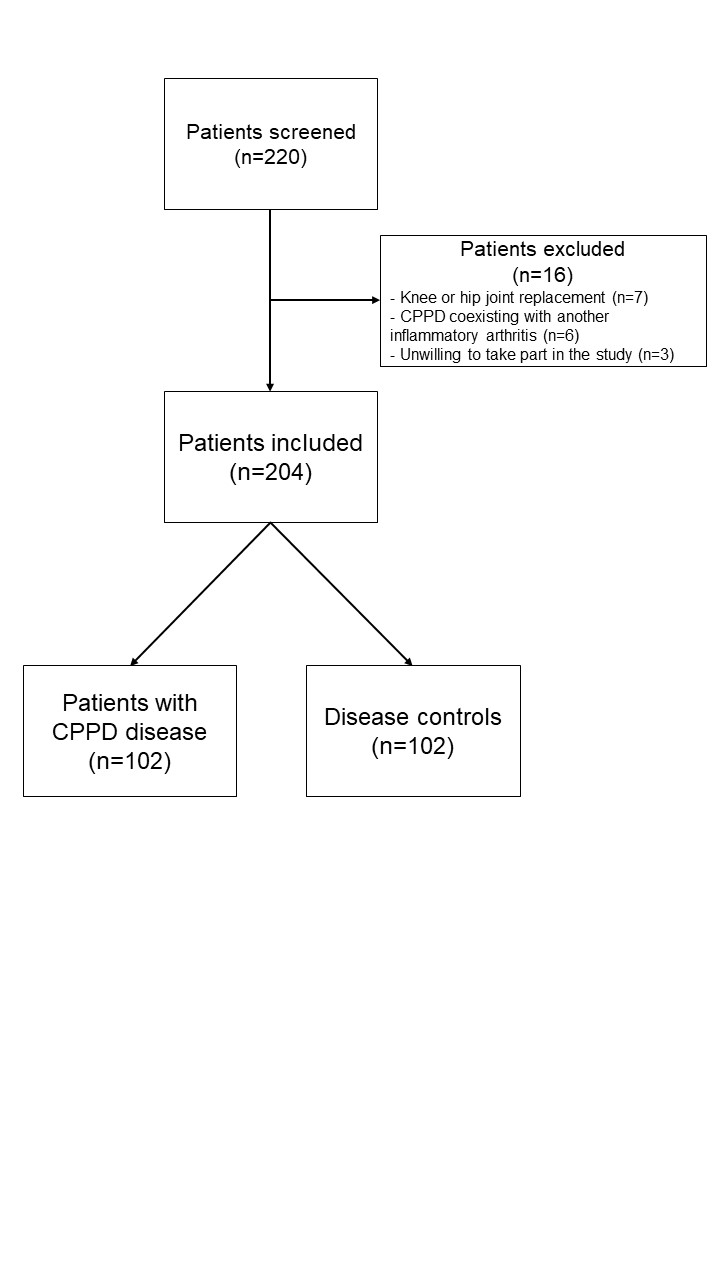


**Supplementary Figure S3.** Proposed US scanning protocol for the diagnosis of CPPD disease.


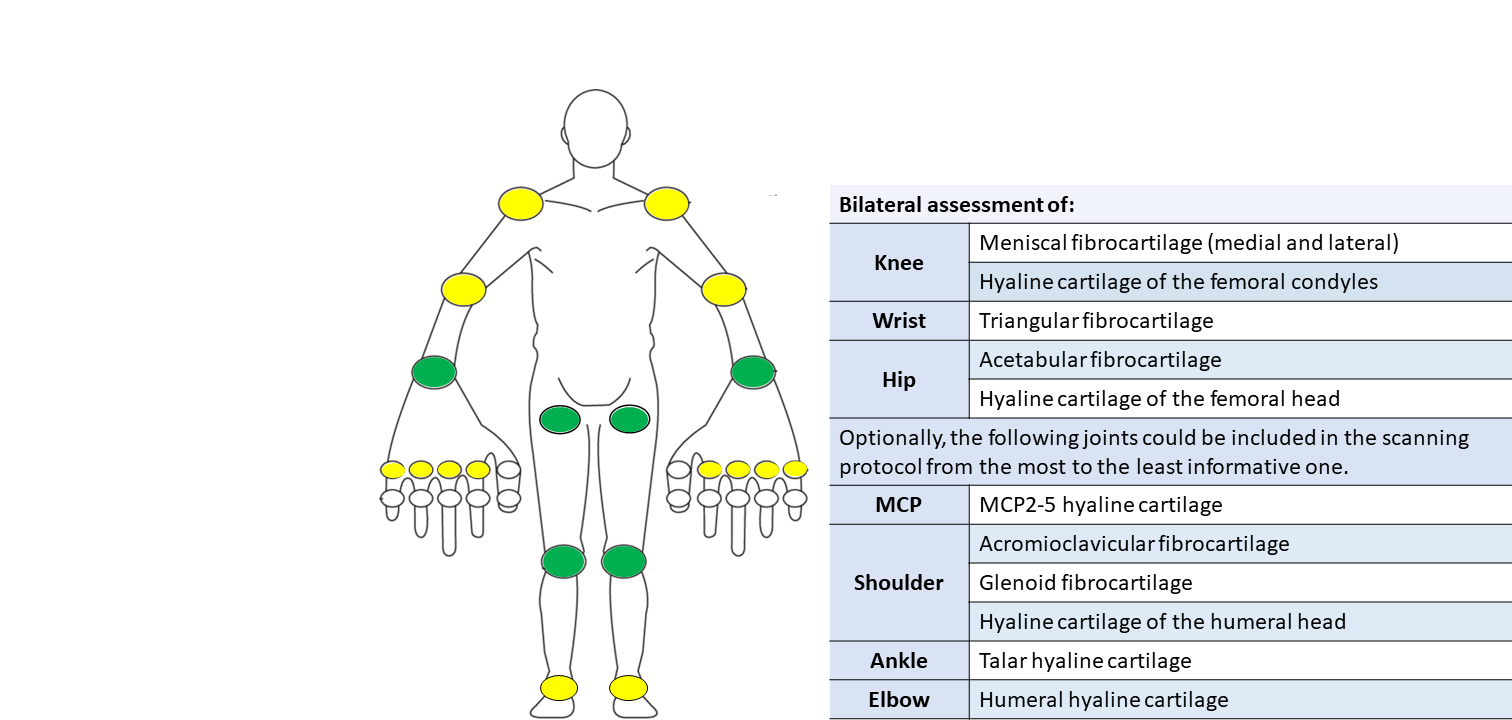

Supplement: kead565_Supplementary_Data [file kead565_supplementary_data.docx]
